# The association between cold exposure and musculoskeletal disorders: a prospective population-based study

**DOI:** 10.1007/s00420-022-01949-2

**Published:** 2023-01-02

**Authors:** Charlotte Lewis, Albin Stjernbrandt, Jens Wahlström

**Affiliations:** Section of Sustainable Health, Department of Public Health and Clinical Medicine, Umeå University, 901 87 Umeå, Sweden

**Keywords:** Occupational exposure, Cold climate, Neck pain, Low back pain, Radiculopathy, Sciatica

## Abstract

**Objectives:**

The aim of the study was to determine the association between occupational ambient cold exposure and neck–shoulder pain (NSP), low back pain (LBP), and radiating LBP.

**Methods:**

The study cohort comprised of 3,843 working subjects in northern Sweden who answered a baseline (spring 2015) and a follow-up questionnaire (spring 2021). NSP, LBP, and radiating LBP were assessed in both surveys. Occupational cold exposure was assessed at baseline, on a whole number numerical rating scale (NRS) and categorized in quartiles. Binary logistic regression determined the association between cold exposure at baseline and incident NSP, LBP, and radiating LBP, adjusted for age, gender, body mass index, smoking, mental stress, and physical workload.

**Results:**

There were statistically significant associations between high occupational ambient cold exposure (NRS 5–7 and NRS 8–10) and NSP (1.59; 95% CI 1.08–2.33 and OR 1.50; 95% CI 1.03–2.19); NRS 8–10 and LBP (OR 1.61; 95% CI 1.13–2.29); and NRS 5–7 and radiating LBP (OR 1.87; 95% CI 1.12–3.16). Gender-stratified analyses showed statistically significant associations between high occupational ambient cold exposure (NRS 5–7 and NRS 8–10) and NSP (OR 1.97; 95% CI 1.07–3.61 and OR 1.97; 95% CI 1.06–3.67) for men and between NRS 8–10 and LBP (OR 1.82; 95% CI 1.14–2.91) and NRS 5–7 and radiating LBP (OR 2.20; 95% CI 1.15–4.24) for women.

**Conclusions:**

Occupational ambient cold exposure was associated with NSP, LBP, and radiating LBP, and should be recognised as a possible occupational risk factor.

**Supplementary Information:**

The online version contains supplementary material available at 10.1007/s00420-022-01949-2.

## Introduction

Low back pain (LBP) and neck–shoulder pain (NSP) are major public health problems, causing work disability, productivity loss, and societal costs (Ekman et al. 2005; Punnett et al. 2005; Ricci et al. 2006; GBD 2017; Safiri et al. 2020). Prevalence (1 month to 1 year) of LBP in the general population has been reported to be 23–50% (Mehlum et al. 2006; Farioli et al. 2014; Yang et al. 2016) with a point prevalence of 7.5% (Wu et al. 2020). The prevalence of lumbar radiculopathy has been estimated to be 3–5% in the general population (Berry et al. 2019), and the incidence 4.7 per 1000 person-years in a military population (Schoenfeld et al. 2012). The prevalence of NSP has been reported at 23–68% (Fejer et al. 2006; Mehlum et al. 2006; Farioli et al. 2014).

There is a large body of occupational epidemiologic findings showing associations between NSP and LBP and several occupational ergonomic exposures, such as manual material handling, awkward body postures, repetitive movements, vibration, and high job demands (Ariens et al. 2000; Marras 2000; Mayer et al. 2012; Farioli et al. 2014; Iqbal and Alghadir 2017; Kuijer et al. 2018; Swedish agency for health technology assessment and assessment of social services 2022). However, it has been suggested that occupational cold exposure could be an additional risk factor for developing NSP and LBP (Pienimäki 2002; Mäkinen and Hassi 2009; Farbu et al. 2021b, a).

Occupational cold exposure has been defined as being subjected to ambient temperatures at or below 10 ℃ (International Organization for Standardization 2008). However, a variety of definitions have been used when assessing cold exposure in scientific papers (Pienimäki 2002). Swedish official statistics report that about 21% of men and 11% of women are occupationally exposed to a cold climate for at least 25% of their working hours (The Swedish Work Environment Authority 2020).

Two reviews from 2002, both concluded the epidemiological evidence for association between cold exposure and NSP and LBP as insufficient, and in need of further research (Hildebrandt et al. 2002; Pienimäki 2002). The scientific base for an association between cold exposure and NSP and LBP has since been growing. Working in cold environment has been associated with LBP in studies investigating cold store workers (OR 2.98; 95% CI 1.3–6.7) as well as miners (OR 1.52; 95% CI 1.30–1.78) (Dovrat and Katz-Leurer 2007; Skandfer et al. 2014). In a recent study by Ghani et al. (2020) the workers in cold storage facilities had a relative risk of neck pain of 15.00 (95% CI 6.33–35.51) compared to their non-cold exposure colleagues. In a study comparing construction workers from regions with different climate within Sweden, working in the coldest region was associated with LBP (OR 1.19; 95% CI 1.14–1.24) and NSP (OR 1.57; 95% CI 1.47–1.67). However, a similar association was observed for office workers and foremen (Burström et al. 2013). Strong associations have also been shown for subjective feeling of being cold and LBP (OR 11.0; 95% CI 4.5–26.8), and NSP (10.5; 95% CI 3.1–35.3) in Norwegian seafood industries (Aasmoe et al. 2008). In a study of Finnish meat processing industries, the occurrence of LBP was the highest among those who experienced “extensive low back cooling” (OR 3.88; 95% CI 1.82–8.25) and prevalence of NSP was highest among those who experienced “extensive neck–shoulder cooling” (OR 6.47; 95% CI 2.79–14.99) (Sormunen et al. 2009a, b). A Swedish cross-sectional study by Stjernbrandt and Farbu (2022), based on the same baseline questionnaire used in the current paper, showed that high occupational ambient cold exposure was associated with NSP (OR 1.36; 95% CI 1.16–1.59), LBP (OR 1.38; 95% CI 1.17–1.63), and lumbar radiculopathy (OR 1.36; 95% CI 1.07–1.73), after adjusting for age, gender, body mass index, physical work load, daily smoking, and mental stress. Similar associations between cold exposure and NSP (OR 1.46; 95% CI 1.13–1.89) was seen in a study on a general working population in northern Norway (Farbu et al. 2019). Prospective studies on cold exposure and NSP and LBP are scarce; however, Farbu et al. (2021a, b) presented an increased risk of having any musculoskeletal complaints in their 7–8-year follow-up of 2,347 working subjects in northern Norway (Incidence rate ratio 1.15; 95% CI 1.03–1.29). Few studies have investigated possible differences between men and women in the association between cold exposure and NSP and LPB, but some have suggested that there might be differences (Sormunen et al. 2009a, b; Stjernbrandt and Hoftun Farbu 2022). The aim of the study was, therefore, to determine the association between occupational ambient cold exposure and neck–shoulder pain, low back pain, and radiating low back pain among male and female individuals of working age.

## Methods

### Study design and setting

**Fig. 1:**
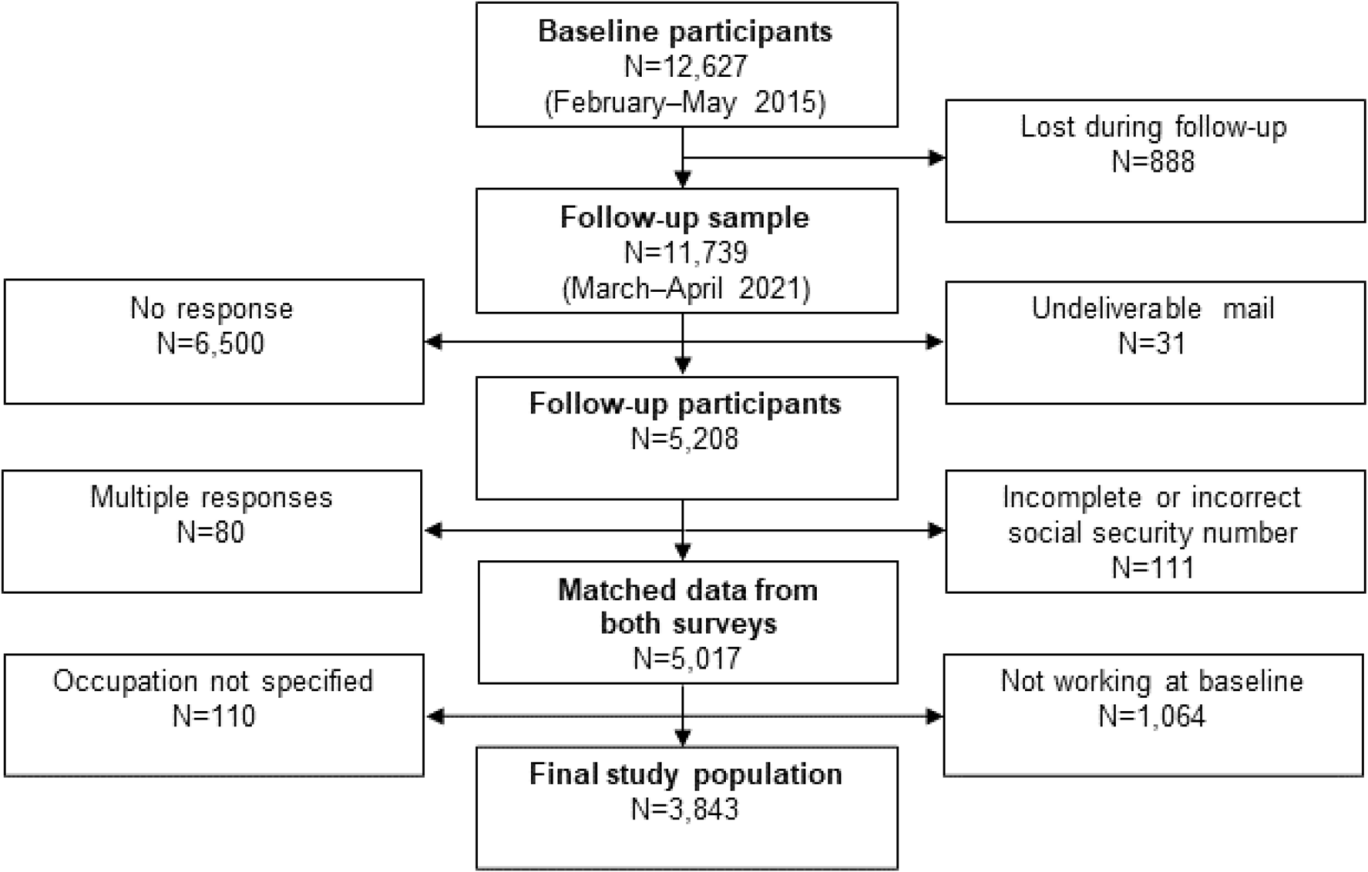
Flow chart, showing the data collection for the follow-up survey based on the participants of the baseline survey. *CHINS* cold and health in northern Sweden

This prospective cohort study was part of the Cold and Health In Northern Sweden (CHINS) research project. Baseline data came from the first postal questionnaire, administered between February and May of 2015, to a sample of 35,144 men and women of working age (18–70 years), in the four northernmost counties in Sweden: Norrbotten, Västerbotten, Västernorrland, and Jämtland. In total, these counties hold a population of approximately 880,000 people and is located between 62°N and 69°N latitude with a mixed subarctic and temperate climate. Follow-up data were assessed by a digital questionnaire between March and April of 2021. All subjects (*N* = 12,627) who had responded to the baseline questionnaire were sent a postal invitation to respond to the follow-up questionnaire, with one postal reminder. In addition, the option to respond to the questionnaire on paper was presented. There were 5,208 responses to the follow-up survey (CHINS2021), yielding a response rate of 44.4%. Due to multiple responses and invalid social security numbers, 191 survey responses could not be matched to the original data set. Subjects who were not working at the time of the baseline survey could not be categorized on occupational cold exposure and were, therefore, excluded (*N* = 1,064), as were participants that had not specified their occupation (*N* = 110). The remaining 3,843 subjects comprised the study cohort available for analysis (Fig. 1). A more detailed description of the data collection can be found in previous publications (Stjernbrandt et al. 2017; Stjernbrandt and Farbu 2022).

### Variables and statistical analyses

Neck–shoulder pain (NSP), low back pain (LBP), and radiating low back pain (radiating LBP) were assessed in both surveys by three questionnaire items: “Do you have pain in neck/shoulders”, “Do you have pain in the lower part of the back”, and “Do you have pain radiating from the back to below the knees (sciatica)”. The answers were given on a four-grade scale, as “none”, “insignificant”, “somewhat”, or “a lot”. Answering “a lot” was considered a positive response. The follow-up survey was used to determine the presence of incident cases (negating pain at baseline and reporting pain at follow-up) to compare them with healthy references, negating pain at both baseline and follow-up. Symptomatic subjects at baseline were excluded from further analyses.

Occupational cold exposure was assessed by the questionnaire described in Stjernbrandt and Farbu (2022): “During work I am exposed to outdoor or cold environments”. The answers were given on whole number numerical rating scales (NRS), ranging from one (“do not agree”) to ten (“fully agree”), and later categorized according to quartiles. Occupation was reported by the respondent, and manually coded in accordance with the International Standard Classification of Occupations (ISCO) (International Labour Organization 2012). A *job–exposure matrix* (JEM) was created, where the physical workload was categorized into *low, medium,* or *high*, based on the two-level ISCO coding. The distribution of ISCO coded occupations were: armed forces (0.5%), skilled agricultural, forestry, and fishery workers (1.7%), elementary occupations (2.6%), self-employed (2.6%), crafts and related trades workers (6.2%), managers (6.7%), plant and machine operators and assemblers (7.5%), clerical support workers (12.4%), technicians and associate professionals (15.5%), service and sales workers (16.0%), and professionals (28.4%). Detailed information of the JEM is described in supplementary data by Stjernbrandt and Farbu (2022). Mental stress was assessed by the questionnaire item “Stress is a condition where you feel tensed, restless, nervous, worried, or have trouble sleeping. Have you experienced such stress during the last month?” (Elo et al. 2003). The answers were dichotomized into *low* (questionnaire responses “none”, “very little”, or “some”) and *high* (questionnaire responses “a lot” or “very much”). Age was categorized into equal spans, body mass index (BMI) by clinically used thresholds for under- and overweight, but both also analyzed continuously, and currently daily smoking dichotomized (yes/no).

Incidence proportion was calculated as the number of incident cases divided by subjects at risk (total sample minus number of subjects with pain at baseline). Yearly incidence proportion was also calculated.

Binary logistic regression was used to determine the association between cold exposure at work and incident NSP, LBP, and radiating LBP, respectively, presented as odds ratios (OR) with 95% confidence intervals (95% CI). Baseline variables used for adjusting were: *age (years)*, *gender (female/male), body mass index (BMI; kg/m*^*2*^*)*, *current daily smoking (yes/no)*, *mental stress (low/high)*, and *physical workload (low/medium/high)*. Pearson Chi Square test was used to determine gender difference in prevalence of NSP, LBP, and radiating LBP. A *p* value < 0.05 was considered statistically significant. Statistical analyses were performed using SPSS (version 27.0, IBM Corporation, Armonk, NY, USA).

## Results

### Descriptive data

The final study population consisted of 2089 women and 1754 men. Other characteristics from the baseline survey are presented in Table 1. NSP was reported by 606 (15.8%) subjects at baseline and 622 (16.2%) subjects at follow-up, LBP was reported by 515 (13.4%) subjects at baseline and 591 (15.4%) at follow-up, and radiating LBP was reported by 225 (5.9%) at baseline and 219 (5.7%) at follow-up (Table 2).

**Table 1:** Baseline information of the study population (*N* = 3843), categorized by occupational cold exposure

Variable	NRS 1		NRS 2–4		NRS 5–7		NRS 8–10	
	*N* = 2111^a^		*N* = 698^a^		*N* = 469^a^		*N* = 497^a^	
	*N*	%	*N*	%	*N*	%	*N*	%
Age (years)								
18–31	408	19.3	140	20.1	107	22.8	129	26.0
32–44	794	37.6	289	41.4	164	35.0	164	33.0
45–57	737	34.9	225	32.2	155	33.0	163	32.8
58–70	172	8.1	44	6.3	43	9.2	41	8.2
Gender								
Male	721	34.2	397	56.9	300	64.0	305	61.4
Female	1390	65.8	301	43.1	169	36.0	192	38.6
Body mass index (kg/m^2^)								
< 20	19	1.0	2	0.3	3	0.7	4	0.9
20–25	885	45.0	266	41.4	160	36.7	170	36.2
25–30	742	37.7	270	42.0	187	42.9	218	46.5
> 30	320	16.3	105	16.3	86	19.7	77	16.4
Physical workload								
Low	1559	73.9	407	58.3	193	41.2	186	37.4
Medium	256	12.1	151	21.6	129	27.5	126	25.4
High	296	14.0	140	20.1	147	31.3	185	37.2
Daily smoking								
No	1992	94.5	664	95.5	437	93.4	465	93.9
Yes	117	5.5	31	4.5	31	6.6	30	6.1
Mental stress								
Low	1627	77.4	555	79.7	360	77.4	400	80.6
High	476	22.6	141	20.3	105	22.6	96	19.4

^a^Exposure data were missing for 68 subjects. *NRS* numerical rating scale

**Table 2:** Prevalence and incidence proportions for neck–shoulder pain (NSP), low back pain (LBP), and radiating low back pain (radiating LBP), for all, and gender separated

Outcome	Measure	Women (*N* = 2089)	Men (*N* = 1754)	All (*N* = 3843)
		*N* (%)	*N* (%)	*N* (%)
NSP	Baseline	420 (20.1)	186 (10.6)	606 (15.8)
	Incident^a^	183/1669 (11.0)	100/1568 (6.4)	283/3237 (8.7)
	Per year^b^	30.5 (1.8)	16.7 (1.1)	47.2 (1.5)
LBP	Baseline	338 (16.2)	177 (10.1)	515 (13.4)
	Incident^a^	186/1751 (10.6)	124/1577 (7.9)	310/3328 (9.3)
	Per year^b^	31 (1.8)	20.5 (1.3)	51.5 (1.5)
Radiating LBP	Baseline	124 (5.9)	101 (5.8)	225 (5.9)
	Incident^a^	81/1956 (4.1)	45/1653 (2.7)	126/3618 (3.5)
	Per year^b^	13.5 (0.7)	7.5 (0.5)	21 (0.6)

^a^Number of incident cases divided by subjects at risk (total sample minus number of subjects with pain at baseline). ^b^Cumulative figure divided by six

### Effects of occupational cold exposure

In the crude analyses on the whole population there were statistically significant associations between high occupational ambient cold exposure (NRS 8–10) and LBP (OR 1.57; 95% CI 1.13–2.18) as well as between NRS 5–7 and radiating LBP (OR 1.82; 95% CI 1.11–2.97) (Table 3). When adjusting for age, gender, smoking, mental stress, and physical workload there were statistically significant associations between high occupational ambient cold exposure (NRS 5–7 and NRS 8–10) and NSP (1.59; 95% CI 1.08–2.33 and OR 1.50; 95% CI 1.03–2.19, respectively), NRS 8–10 and LBP (OR 1.61; 95% CI 1.13–2.29) and NRS 5–7 and radiating LBP (OR 1.87; 95% CI 1.12–3.16) (Table 3).

**Table 3:** Binary logistic regression for incident neck–shoulder pain (NSP), low back pain (LBP), and radiating LBP, among working subjects (*N* = 3843)

	NSP				LBP				Radiating LBP			
	Incident	Healthy references	OR (95% CI)^a^	OR (95% CI)	Incident	Healthy references	OR (95% CI) ^a^	OR (95% CI)	Incident	Healthy references	OR (95% CI)^a^	OR (95% CI)
All occupational cold exposure *N* (%)												
NRS 1	146 (52.5)	1617 (56.8)	–	–	156 (51.7)	1644 (57.0)	–	–	62 (51.2)	1915 (56.9)	–	–
NRS 2–4	44 (15.8)	532 (18.7)	0.92 (0.65–1.30)	1.03 (0.72–1.48)^b^	53 (17.5)	550 (19.1)	1.02 (0.73–1.41)	1.09 (0.78–1.53)^b^	15 (12.4)	635 (18.9)	0.73 (0.41–1.29)	0.79 (0.44–1.42)^b^
NRS 5–7	43 (15.5)	337 (11.8)	1.41 (0.99–2.03)	1.59 (1.08–2.33)*^b^	39 (12.9)	326 (11.3)	1.26 (0.87–1.83)	1.37 (0.93–2.03)^b^	23 (19.0)	391 (11.6)	1.82 (1.11–2.97)*	1.87 (1.12–3.16)*^b^
NRS 8–10	45 (16.2)	360 (12.6)	1.38 (0.97–1.97)	1.50 (1.03–2.19)*^b^	54 (17.9)	363 (12.6)	1.57 (1.13–2.18)*	1.61 (1.13–2.29)*^b^	21 (17.4)	425 (12.6)	1.53 (0.92–2.53)	1.43 (0.82–2.49)^b^
Men occupational cold exposure *N* (%)												
NRS 1	26 (26.3)	630 (44.5)	–	–	41 (33.9)	605 (43.5)	–	–	14 (31.8)	667 (42.9)	–	–
NRS 2–4	21 (21.2)	324 (22.9)	1.57 (0.87–2.83)	1.41 (0.77–2.57)^c^	30 (24.8)	327 (23.5)	1.35 (0.83–2.21)	1.39 (0.84–2.28)^c^	5 (11.4)	371 (23.9)	0.64 (0.23–1.80)	0.57 (0.20–1.64)^c^
NRS 5–7	25 (25.3)	234 (16.5)	2.59 (1.47–4.57)*	1.97 (1.07–3.61)*^c^	24 (19.8)	224 (16.1)	1.58 (0.93–2.68)	1.64 (0.94–2.88)^c^	10 (22.7)	256 (16.5)	1.86 (0.82–4.24)	1.61 (0.67–3.91)^c^
NRS 8–10	27 (27.3)	228 (16.1)	2.87 (1.64–5.02)*	1.97 (1.06–3.67)*^c^	26 (21.5)	234 (16.8)	1.64 (0.98–2.74)	1.59 (0.89–2.81)^c^	15 (34.1)	260 (16.7)	2.75 (1.31–5.77)*	1.95 (0.83–4.56)^c^
Women occupational cold exposure *N* (%)												
NRS 1	120 (67.0)	987 (69.0)	–	–	115 (63.5)	1039 (69.6)	–	–	48 (62.3)	1248 (68.9)	–	–
NRS 2–4	23 (12.8)	208 (14.5)	0.91 (0.57–1.46)	0.88 (0.54–1.41)^c^	32 (17.7)	274 (18.4)	0.93 (0.58–1.49)	0.89 (0.55–1.43)^c^	10 (13.0)	264 (14.6)	0.99 (0.49–1.97)	0.96 (0.48–1.94)^c^
NRS 5–7	18 (10.1)	103 (7.2)	1.44 (0.84–2.46)	1.38 (0.80–2.40)^c^	34 (18.8)	180 (12.1)	1.33 (0.75–2.36)	1.23 (0.68–2.21)^c^	13 (16.9)	135 (7.5)	2.50 (1.32–4.74)*	2.20 (1.15–4.24)*^c^
NRS 8–10	18 (10.1)	132 (9.2)	1.12 (0.66–1.90)	1.07 (0.63–1.84)^c^			1.96 (1.25–3.08)*	1.82 (1.14–2.91)*^c^	6 (7.8)	165 (9.1)	0.95 (0.40–2.24)	0.91 (0.38–2.17)^c^

*OR* odds ratio, *NRS* numerical rating scale, *95% CI* ninety-five percent confidence interval

*Significant at the 0.05 level

^a^Crude estimate. ^b^Adjusted for gender, continuous body mass index, continuous age, physical workload, daily smoking, and mental stress. ^c^Adjusted for continuous body mass index, continuous age, physical workload, daily smoking, and mental stress

### Gender differences

There were more women than men reporting NSP (19.9% vs. 11.7%, *p* < 0.001), LBP (17.4% vs. 13.0%, *p* < 0.001) and radiating LBP (6.4% vs. 4.8%, *p* = 0.035) at follow-up. There were more female incident cases for both NSP (8.8% vs. 5.7%, *p* < 0.001), LBP (8.9% vs. 7.1%, *p* = 0.006), and radiating LBP (3.9% vs. 2.6%, *p* = 0.021).

When stratifying on gender, there were statistically significant crude associations between high occupational ambient cold exposure (NRS 5–7 and NRS 8–10) and NSP (OR 2.59; 95% CI 1.47–4.57 and OR 2.87; 95% CI 1.64–5.02, respectively) and NRS 8–10 and radiating LBP (OR 2.75; 95% CI 1.31–5.77) for men (Table 3). The association for NSP was still present when adjusting for possible confounders (OR 1.97; 95% CI 1.07–3.61 and OR 1.97; 95% CI 1.06–3.67, respectively). For women, statistically significant crude associations were found between NRS 8–10 and LBP (OR 1.96; 95% CI 1.25–3.08), and NRS 5–7 and radiating LBP (OR 2.50; 95% CI 1.32–4.74). When adjusting for confounders, the association was still present (LBP OR 1.82; 95% CI 1.14–2.91 and radiating LBP OR 2.20; 95% CI 1.15–4.24).

The full logistic regression model with all variables is presented in Online resource 1–3.

## Discussion

### Main findings

High occupational ambient cold exposure was associated with NSP, LBP, and radiating LBP. When stratifying on gender, associations were observed between occupational ambient cold exposure and NSP among men, and LBP and radiating LBP among women.

Similar associations between cold exposure and long lasting (> 3 months) NSP (OR 1.46; 95% CI 1.13–1.89) was seen in a study on a general working population in northern Norway (Farbu et al. 2019), adjusting for age, gender, smoking, educational level, physical activity level, and insomnia, while they did not find any associations with LBP (OR 1.18, 95% CI 0.91–1.52). A longitudinal study based on the same sample found that working in a cold environment at baseline was associated with MSDs lasting three months or more, 7–8 years later (incidence rate ratio 1.15; 95% CI 1.03–1.29) (Farbu et al. 2021a, b). However, anatomical location was not specified. A Swedish study compared construction workers from regions with different climate in Sweden and found that working in the coldest region was associated with NSP (OR 1.57: 95% CI 1.47–1.67) as well as LBP (OR 1.19: 95% CI 1.14–1.24) (Burström et al. 2013). In a study on Russian mine workers, working in cold conditions (< 10 °C) was associated with reporting LBP (OR 1.82; 95% CI 1.55–2.15), after adjusting for gender, BMI, duration of work, physical fitness level, and stress (Skandfer et al. 2014).

Furthermore, working in cold indoor environments has also been associated with MSDs. A study on Israeli male cold store workers, exposed to indoor temperatures around − 20 °C, had an increased risk of back pain (OR 2.9; 95% CI 1.3–6.7) compared to their unexposed colleagues who performed similar tasks (Dovrat and Katz-Leurer 2007). A study on Pakistani workers compared those working in cold storage facilities with indoor temperatures ranging from − 20 to − 30 ºC with those who did not have any exposure to cold indoor environments and found a 15-times increased risk of repeated NSP or upper extremities (RR 15.00; 95% CI 6.33–35.51) (Ghani et al. 2020). There were, however, differences in physical load between the cold-exposed and unexposed groups that could explain part of the increased risk. A Norwegian study on people working in the seafood industry found strong associations for the subjective feeling of being cold and LBP (OR 11.0; 95% CI 4.5–26.8), and NSP (10.5; 95% CI 3.1–35.3) (Aasmoe et al. 2008). Subjectively feeling cold was associated with both NSP and LBP in a study of Finnish meat processing industries (Sormunen et al. 2009a, b). Those who experienced “extensive low back cooling” had the highest risk of LBP (OR 3.88; 95% CI 1.82–8.25) and those who experienced “extensive neck–shoulder cooling” had an increased risk of NSP (OR 6.47; 95% CI 2.79–14.99) (Sormunen et al. 2009a, b).

The prevalence of NSP at baseline was 15.8% in the whole population (women 20.1%; men 10.6%), which is lower than the official Swedish statistics, reporting that 45% of the women and 25% of the men have pain in the neck and the upper back (The Swedish Work Environment Authority 2020). This difference is probably due to the cutoff for defining pain used in the present study with only the highest category (four-grade scale, as “none”, “insignificant”, “somewhat”, or “a lot”). When changing the cutoff and including the “somewhat” category the baseline prevalence was 40.2%, closely resembling the national statistics. The reason for using the higher cutoff was to ascertain that the case group included individuals with more severe symptoms. Another explanation to the difference in prevalence rates could be that we used a more defined anatomic location in our survey, i.e., not including the upper part of the back. Fejer et al. (2006) reported a point prevalence of neck pain more in line with ours, ranging from 5.9% to 22.2% in the adult population (15–74 years), while Safiri et al. (2020) reported a point prevalence in western Europe as low as 4.6%. However, in the latter study, no age restrictions were applied, nor criteria on being currently working.

The prevalence of LBP at baseline was 13.4% (women 16.2%; men 10.1%), which is in line with Ihlebaek et al. (2006) reporting a point prevalence of 13.4% (women 16.8%; men 9.9%) in southern Norway and slightly lower than the 18.2% (women 20.4%; men 14.6%) in southern Sweden. In addition, a systematic review of global prevalence of LBP presented a mean point prevalence of 11.9% (Hoy et al. 2012), which is well in line with our results.

The prevalence of radiating LBP at baseline was 5.9% (women 5.9%; men 5.8%) which is in the same range as have been reported earlier (Berry et al. 2019), but substantially higher than what was reported by Younes et al. (2006) who studied a Tunisian urban population with a point prevalence of 0.75% and the review by Konstantinou and Dunn (2008) that reported a point prevalence of 1.6%. The difference in prevalence of radiating LBP is believed to be explained by differences in sampling and case definitions (i.e., demanding radicular pain below the hip or knee).

The annual incident proportion of NSP in our study was 1.5% (women 1.8%; men 1.1%), for LBP 1.5% (women 1.8%, men 1.3%), and radiating LBP 0.6% (women 0.7%, men 0.5%), highlighting the magnitude of the problem.

### Gender

We found gender differences in NSP, LBP, and radiating LBP prevalence at baseline and follow-up as well as regarding incidence proportion. There were also gender differences in occupational ambient cold exposure with a smaller proportion of women being highly exposed (9.4% of women vs. 17.7% of men in NRS 8–10). Similar gender differences in prevalence of both NSP and LBP in the Swedish population have been shown by others (Bingefors and Isacson 2004; Wahlstedt et al. 2010) and several papers have reported a higher prevalence of musculoskeletal pain in general in women compared to men (Bingefors and Isacson 2004; Treaster and Burr 2004; Fillingim et al. 2009; Leboeuf-Yde et al. 2009; Leijon et al. 2009).

The gender stratified analysis showed that there were differences in the associations between ambient occupational cold exposure and the different pain outcomes for men and for women. For men, a significant association was found only between cold exposure and NSP, while there were significant associations between cold exposure and LBP and radiating LBP for women. Since the prevalence of NSP was substantially higher among women than men, the rather small added proportion related to occupational cold exposure might not have been discernable. Furthermore, we adjusted for physical exposures at work with a simple JEM, and it cannot be ruled out that there were gender differences that a more detailed physical exposure assessment could have revealed. Finally, due to low numbers of highly cold exposed females, associations with our outcomes for women could be hard to detect in our cohort.

The mechanisms for gender differences in the susceptibility for cold-induced discomfort are not clear. Sormunen et al. (2009a, b) reported that female workers experienced cold ambient temperature, and other environmental factors as significantly more harmful than their male counterparts. Pienimäki et al. (2014) pointed out that women may have a lower temperature threshold for reporting symptoms. In contrast, in an experimental study, working in cold, compared with thermoneutral conditions, increased muscular activity in the forearm and upper arm extensors only in men and not in women (Sormunen et al. 2009a, b).

Personal protective equipment (PPE), such as heavy caps, coats, and gloves can be used to protect workers from ambient cold exposure. However, it may alter working posture and hinder movements, thus increasing the physical workload (Piedrahita et al. 2004; Dovrat and Katz-Leurer 2007). In addition, gender differences in access to, as well as usage of PPE cannot be ruled out.

To conclude, further studies are needed to elucidate mechanisms behind gender differences regarding effects of cold work.

### Mechanisms

The mechanism for cold-induced MSDs is not established, but different explanations have been suggested. An increase in muscle activation as well as a reduction in muscle activation gaps, due to exposure to moderately cold conditions in several upper extremity muscles during repetitive work in ambient temperatures of 4–10 °C has been shown (Oksa 2002; Oksa et al. 2002, 2006, 2012; Piedrahita et al. 2008; Sormunen et al. 2009a, b; Renberg et al. 2020a, b). Cold exposure has not been shown to have any effect on muscle activation in isometric muscle work. Some studies have even showed a beneficial effect, where the endurance time was increased and the rate of fatigue slower when muscle temperature was below normal but higher than 27 °C (Oksa 2002; Renberg et al. 2020a, b). On the other hand, working in cold environments may reduce the temperature of the working muscle tissue and slow nerve conduction velocity which in turn could be seen as a shift to lower frequencies in the frequency component of EMG (Petrofsky and Laymon 2005).

Cold-induced vasospasm has been found in 20% of patients with chronic LBP and in 38% of patients with fibromyalgia but in only 8% of healthy people (Lapossy et al. 1994). Thus, another plausible mechanism could be that reduced muscular blood flow induces ischemic nociceptive pain during situations with high physical demands. In support of this view, a significantly lower blood flow was seen in a laboratory study, during wrist flexion–extension repetitive work in two cold conditions (5 °C) compared to during a thermoneutral condition (25 °C) (Oksa et al. 2002). Finally, a study found that workers with chronic pain reported more indoor climate complaints than pain-free controls despite similar actual indoor climate and concluded that the difference was likely due to central sensitization (Sundstrup et al. 2015).

For lumbar disc disease, it has been suggested that cooling is unfavorable for the diffusion of the intervertebral disc fluid when combined with heavy work and static postures (Hildebrandt et al. 2002).

### Methodological limitations

The rather low response rate may have introduced a sampling bias that was not controlled for. In addition, separating the exposure measure into four categories and stratifying for gender reduced the statistical power and increased the risk for type 2 error. The measure of cold exposure was subjectively reported, arbitrarily scaled, and could not be translated into exposure intensity or duration. In addition, the cut points for cold exposure were data-driven and not based on any a priori knowledge about physiological threshold effects. Furthermore, potential effects of leisure-time cold exposure were not investigated in this study. However, a previous cross-sectional study on the same cohort revealed no effect of exposure occurring outside of work (Stjernbrandt and Farbu 2022). In clinical practice, there is likely a rather large overlap between reporting LBP and radiating LBP. However, in our study we strived to separate these two entities, by defining them in two separate questions in the questionnaire. Separating LBP and radiating LBP based on subjective reporting of symptoms might not be motivated from a clinical standpoint and makes the interpretation of the results more challenging. However, the separation was motivated by the assumption that pathophysiological mechanisms may differ in the sense that radiating LBP is less related to cold-related effects on postural muscle activity and more associated with degenerative changes in intervertebral discs and adjacent joint and bony structures. Another limitation is the fact that the duration of pain was not investigated in our study. There might be differences in short-term and more chronic conditions regarding the effects of cold exposure. Furthermore, the JEM employed in the current study was based on the major and sub-major ISCO groups and resulted in a rather crude measure of physical workload. A more detailed JEM would have improved the assessment of the physical load. Finally, since the study focused on currently working subjects, there is a risk of a healthy worker effect which might have attenuated the effect sizes.

### Methodological strengths

One major strength of our study is the fact that it was population-based and utilized a prospective approach. To the authors’ knowledge, only one previous study on occupational cold exposure and MSDs has been performed, and that study specified outcomes as single- or multi-site musculoskeletal complaints without any details on anatomical region (Farbu et al. 2021a, b). The present study was conducted in a subarctic and temperate climate, where manual work is common, and this was a suitable setting for a study aiming to explore effects of cold exposure on MSDs. Since both prevalence and mechanistic explanations may differ, reporting gender-stratified results was also relevant. The study collected ample data on known confounding factors to allow for adjusted regression models.

## Conclusions

To conclude, ambient occupational cold exposure was associated with NSP, LBP, and radiating LBP, and should be recognised as a possible occupational risk factor. The association might differ between men and women. Further studies, with a higher resolution of the assessment of both cold exposure and adjusting factors such as physical workload are needed. In addition, the interaction between cold and other risk factors should be investigated.

## Supplementary Information

Below is the link to the electronic supplementary material.

Supplementary file1 (DOCX 28 kb)

## Data Availability

Source data can be made available upon personal request.
