# Supplementary material for: The association between cold exposure and musculoskeletal disorders: a prospective population-based study
Source: Int Arch Occup Environ Health. 2023 Jan 2;96(4):565–75. doi: 10.1007/s00420-022-01949-2 (PMC10079729; doi:10.1007/s00420-022-01949-2)
Supplement: Supplementary file 1 — Supplementary file1 (DOCX 28 kb) [file 420_2022_1949_MOESM1_ESM.docx]

Online Resource 1. Binary logistic regression for incident neck-shoulder pain (NSP), low back pain (LBP), and radiating low back pain (radiating LBP), among working subjects (N= 3,843). All adjusting factors presented.

|  | | **NSP** | | | | **LBP** | | | | **Radiating LBP** | | | |
| --- | --- | --- | --- | --- | --- | --- | --- | --- | --- | --- | --- | --- | --- |
|  | |  |  |  |  |  |  |  |  |  |  |  |  |
| Factor | | Incident | Healthy references | OR (95% CI) ^a^ | OR (95% CI) ^b^ | Incident | Healthy references | OR (95% CI) ^a^ | OR (95% CI) ^b^ | Incident | Healthy references | OR (95% CI) ^a^ | OR (95% CI) ^b^ |
| Occupational cold exposure | |  |  |  |  |  |  |  |  |  |  |  |  |
| N (%) | NRS 1 | 146 (52.5) | 1,617 (56.8) | - | - | 156 (51.7) | 1,644 (57.0) | - | - | 62 (51.2) | 1,915 (56.9) | - | - |
|  | NRS 2–4 | 44 (15.8) | 532 (18.7) | 0.92 (0.65–1.30) | 1.03 (0.72–1.48) | 53 (17.5) | 550 (19.1) | 1.02 (0.73–1.41) | 1.09 (0.78–1.53) | 15 (12.4) | 635 (18.9) | 0.73 (0.41–1.29) | 0.79 (0.44–1.42) |
|  | NRS 5–7 | 43 (15.5) | 337 (11.8) | 1.41 (0.99–2.03) | 1.59 (1.08–2.33) * | 39 (12.9) | 326 (11.3) | 1.26 (0.87–1.83) | 1.37 (0.93–2.03) | 23 (19.0) | 391 (11.6) | 1.82 (1.11–2.97) * | 1.87 (1.12–3.16) * |
|  | NRS 8–10 | 45 (16.2) | 360 (12.6) | 1.38 (0.97–1.97) | 1.50 (1.03–2.19) * | 54 (17.9) | 363 (12.6) | 1.57 (1.13–2.18) | 1.61 (1.13–2.29) | 21 (17.4) | 425 (12.6) | 1.53 (0.92–2.53) | 1.43 (0.82–2.49) |
| Age, mean (SD) | | 47.6 (10.9) | 49.9 (11.3) |  | 0.99 (0.98-1.00) * | 49.2 (10.7) | 49.7 (11.3) |  | 1.00 (0.99-1.01) | 52.5 (9.4) | 49.6 (11.3) |  | 1.03 (1.01-1.05) * |
| Gender | |  |  |  |  |  |  |  |  |  |  |  |  |
| N (%) | Male | 100 (35.3) | 1,436 (49.8) |  | - | 124 (40.0) | 1,408 (48.3) |  | - | 45 (35.7) | 1,570 (46.1) |  | - |
|  | Female | 183 (64.7) | 1,447 (50.2) |  | 1.96 (1.49-2.59) * | 186 (60.0) | 1,510 (51.7) |  | 1.51 (1.16–1.96) * | 81 (64.3) | 1,832 (53.9) |  | 1.94 (1.28–2.94) * |
| Body mass index (kg/m²), mean (SD) | | 26.4(5.0) | 26.0 (4.3) |  | 1.03 (1.00–1.05) | 26.5 (4.8) | 25.9 (4.4) |  | 1.03 (1.00–1.05) * | 27.5 (4.8) | 26.0 (4.5) |  | 1.05 (1.01–1.08) * |
| Physical workload ^c^ | |  |  |  |  |  |  |  |  |  |  |  |  |
| N (%) | Low | 162 (57.2) | 1,838 (63.8) |  | - | 186 (60.0) | 1,860 (63.7) |  | - | 60 (47.6) | 2,162 (63.6) |  | - |
|  | Medium | 55 (19.4) | 488 (16.9) |  | 1.37 (0.97–1.94) | 55 (17.7) | 498 (17.1) |  | 1.12 (0.80–1.57) | 30 (23.8) | 576 (16.9) |  | 1.98 (1.23–3.20) * |
|  | High | 66 (23.3) | 557 (19.3) |  | 1.23 (0.89–1.70) | 69 (22.3) | 560 (19.2) |  | 1.15 (0.84–1.58) | 36 (28.6) | 664 (19.5) |  | 1.70 (1.08–2.69) * |
| Daily smoking | |  |  |  |  |  |  |  |  |  |  |  |  |
| N (%) | No | 265 (93.6) | 2,742 (95.3) |  | - | 294 (95.5) | 2,773 (95.2) |  | - | 113 (89.7) | 3,234 (95.2) |  | - |
|  | Yes | 18 (6.4) | 135 (4.7) |  | 1.25 (0.73–2.13) | 14 (4.5) | 140 (4.8) |  | 0.88 (0.50–1.56) | 13 (10.3) | 162 (4.8) |  | 1.56 (0.83–2.95) |
| Mental stress | |  |  |  |  |  |  |  |  |  |  |  |  |
| N (%) | Low | 204 (72.3) | 2,398 (83.5) |  | - | 216 (69.9) | 2,401 (82.5) |  | - | 89 (70.6) | 2,702 (79.7) |  | - |
|  | High | 78 (27.7) | 474 (16.5) |  | 1.78 (1.33–2.38) * | 93 (30.1) | 508 (17.5) |  | 1.98 (1.51–2.58) * | 37 (29.4) | 687 (20.3) |  | 1.70 (1.12–2.57) * |

*OR* odds ratio, *NRS* numerical rating scale, *95% CI* ninety-five percent confidence interval.

* Significant at the 0.05 level.

^a^ Crude estimate. ^b^ Adjusted for gender, continuous body mass index, continuous age, physical workload, daily smoking and mental stress. ^c^ *Low* indicates sedentary work, *medium* ambulatory work, and *high* activities such as heavy lifting and climbing.

Online Resource 2. Binary logistic regression for incident neck-shoulder pain (NSP), low back pain (LBP), and radiating low back pain (radiating LBP) among female working subjects (N=2,089).

|  | | **NSP** | | | | **LBP** | | | | **Radiating LBP** | | | |
| --- | --- | --- | --- | --- | --- | --- | --- | --- | --- | --- | --- | --- | --- |
|  | |  |  |  |  |  |  |  |  |  |  |  |  |
| Factor | | Incident | Healthy references | OR (95% CI) ^a^ | OR (95% CI) ^b^ | Incident | Healthy references | OR (95% CI) ^a^ | OR (95% CI) ^b^ | Incident | Healthy references | OR (95% CI) ^a^ | OR (95% CI) ^b^ |
| Occupational cold exposure | |  |  |  |  |  |  |  |  |  |  |  |  |
| N (%) | NRS 1 | 120 (67.0) | 987 (69.0) |  | - | 115 (63.5) | 1,039 (69.6) | - | - | 48 (62.3) | 1,248 (68.9) | - | - |
|  | NRS 2–4 | 23 (12.8) | 208 (14.5) | 0.91 (0.57–1.46) | 0.88 (0.54–1.41) | 32 (17.7) | 274 (18.4) | 0.93 (0.58–1.49) | 0.89 (0.55–1.43) | 10 (13.0) | 264 (14.6) | 0.99 (0.49–1.97) | 0.96 (0.48–1.94) |
|  | NRS 5–7 | 18 (10.1) | 103 (7.2) | 1.44 (0.84–2.46) | 1.38 (0.80–2.40) | 34 (18.8) | 180 (12.1) | 1.33 (0.75–2.36) | 1.23 (0.68–2.21) | 13 (16.9) | 135 (7.5) | 2.50 (1.32–4.74) * | 2.20 (1.15–4.24) * |
|  | NRS 8–10 | 18 (10.1) | 132 (9.2) | 1.12 (0.66–1.90) | 1.07 (0.63–1.84) |  |  | 1.96 (1.25–3.08) * | 1.82 (1.14–2.91) * | 6 (7.8) | 165 (9.1) | 0.95 (0.40–2.24) | 0.91 (0.38–2.17) |
| Age, (years) mean (SD) | | 45.0 (10.9) | 48.8 (11.4) |  | 0.98 (0.96–0.99) * | 47.7 (10.8) | 48.5 (11.5) |  | 0.99 (0.98–1.01) | 50.1 (9.8) | 48.3 (11.5) |  | 1.02 (1.00–1.04) |
| Body mass index (kg/m²), mean (SD) | | 25.7 (5.6) | 25.3 (4.7) |  | 1.03 (1.00–1.06) * | 26.7 (5.7) | 25.2 (4.7) |  | 1.04 (1.01–1.07) * | 27.0 (5.3) | 25.3 (4.8) |  | 1.04 (1.00–1.08) |
| Physical work load^c^ | |  |  |  |  |  |  |  |  |  |  |  |  |
| N (%) | Low | 126 (68.9) | 1,023 (70.7) |  | - | 120 (64.5) | 1,072 (71.0) |  | - | 44 (54.3) | 1,289 (70.4) |  | - |
|  | Medium | 25 (13.7) | 144 (10.0) |  | 1.22 (0.75–1.99) | 26 (14.0) | 155 (10.3) |  | 1.42 (0.89–2.28) | 14 (17.3) | 197 (10.8) |  | 1.83 (0.96–3.51) |
|  | High | 32 (17.5) | 280 (19.4) |  | 0.85 (0.55–1.31) | 40 (21.5) | 283 (18.7) |  | 1.21 (0.81–1.80) | 23 (28.4) | 346 (18.9) |  | 1.74 (1.01–3.00) * |
| Daily smoking | |  |  |  |  |  |  |  |  |  |  |  |  |
| N (%) | No | 174 (95.1) | 1,360 (94.2) |  | - | 174 (93.5) | 1,424 (94.5) |  | - | 71 (87.7) | 1,724 (94.3) |  | - |
|  | Yes | 9 (4.9) | 84 (5.8) |  | 0.90 (0.44–1.85) | 12 (6.5) | 83 (5.5) |  | 1.10 (0.58–2.09) | 10 (12.3) | 105 (5.7) |  | 1.89 (0.92–3.88) |
| Mental stress | |  |  |  |  |  |  |  |  |  |  |  |  |
| N (%) | Low | 121 (66.5) | 1,163 (80.7) |  | - | 120 (64.9) | 1,196 (79.4) |  | - | 53 (65.4) | 1,376 (75.4) |  | - |
|  | High | 61 (33.5) | 279 (19.3) |  | 1.96 (1.39–2.77) * | 65 (35.1) | 310 (20.6) |  | 2.03 (1.45–2.84) * | 28 (34.6) | 450 (24.6) |  | 1.75 (1.07–2.84) * |

*OR* odds ratio, *NRS* numerical rating scale, *95% CI* ninety-five percent confidence interval.

* Significant at the 0.05 level.

^a^ Crude estimate. ^b^ Adjusted for gender, continuous body mass index, continuous age, physical workload, daily smoking, and mental stress. ^c^ *Low* indicates sedentary work, *medium* ambulatory work, and *high* activities such as heavy lifting and climbing.

Online Resource 3. Binary logistic regression for incident neck-shoulder pain (NSP), low back pain (LBP), and radiating low back pain (radiating LBP) among male working subjects (N=1,754).

|  | | **NSP** | | | | **LBP** | | | | **Radiating LBP** | | | |
| --- | --- | --- | --- | --- | --- | --- | --- | --- | --- | --- | --- | --- | --- |
|  | |  |  |  |  |  |  |  |  |  |  |  |  |
| Factor | | Incident | Healthy references | OR (95% CI) ^a^ | OR (95% CI) ^b^ | Incident | Healthy references | OR (95% CI) ^a^ | OR (95% CI) ^b^ | Incident | Healthy references | OR (95% CI) ^a^ | OR (95% CI) ^b^ |
| Occupational cold exposure | |  |  |  |  |  |  |  |  |  |  |  |  |
| N (%) | NRS 1 | 26 (26.3) | 630 (44.5) | - | - | 41 (33.9) | 605 (43.5) | - | - | 14 (31.8) | 667 (42.9) | - | - |
|  | NRS 2–4 | 21 (21.2) | 324 (22.9) | 1.57 (0.87–2.83) * | 1.41 (0.77–2.57) | 30 (24.8) | 327 (23.5) | 1.35 (0.83–2.21) | 1.39 (0.84–2.28) | 5 (11.4) | 371 (23.9) | 0.64 (0.23–1.80) | 0.57 (0.20–1.64) |
|  | NRS 5–7 | 25 (25.3) | 234 (16.5) | 2.59 (1.47–4.57) * | 1.97 (1.07–3.61) * | 24 (19.8) | 224 (16.1) | 1.58 (0.93–2.68) | 1.64 (0.94–2.88) | 10 (22.7) | 256 (16.5) | 1.86 (0.82–4.24) * | 1.61 (0.67–3.91) |
|  | NRS 8–10 | 27 (27.3) | 228 (16.1) | 2.87 (1.64–5.02) * | 1.97 (1.06–3.67) * | 26 (21.5) | 234 (16.8) | 1.64 (0.98–2.74) | 1.59 (0.89–2.81) | 15 (34.1) | 260 (16.7) | 2.75 (1.31–5.77) * | 1.95 (0.83–4.56) |
| Age, (years) mean (SD) | | 49.6 (11.2) | 50.4 (11.3) |  | 1.00 (0.98–1.02) | 50.4 (10.2) | 50.4 (11.4) |  | 1.00 (0.99–1.02) | 55.8 (6.9) | 50.3 (11.3) |  | 1.06 (1.03–1.10) * |
| Body mass index (kg/m²), mean (SD) | | 27.0 (3.6) | 26.5 (3.8) |  | 1.02 (0.97–1.08) | 26.5 (3.3) | 26.5 (3.8) |  | 0.99 (0.94–1.04) | 27.9 (3.9) | 26.5 (3.8) |  | 1.11 (1.03–1.19) * |
| Physical work load ^c^ | |  |  |  |  |  |  |  |  |  |  |  |  |
| N (%) | Low | 36 (36.0) | 815 (56.8) |  | - | 66 (53.2) | 788 (56.0) |  | - | 16 (35.6) | 873 (55.6) |  | - |
|  | Medium | 300 (30.0) | 344 (24.0) |  | 1.64 (0.96–2.81) | 29 (23.4) | 343 (24.4) |  | 0.91 (0.55–1.48) | 16 (35.6) | 379 (24.1) |  | 1.84 (0.85–3.99) |
|  | High | 34 (34.0) | 277 (19.3) |  | 2.12 (1.23–3.67) * | 29 (23.4) | 277 (19.7) |  | 1.07 (0.64–1.79) | 13 (28.9) | 318 (20.3) |  | 1.48 (0.62–3.56) |
| Daily smoking | |  |  |  |  |  |  |  |  |  |  |  |  |
| N (%) | No | 91 (91.0) | 1,382 (96.4) |  | - | 120 (98.4) | 1,349 (95.9) |  | - | 42 (93.3) | 1,510 (96.4) |  | - |
|  | Yes | 9 (9.0) | 51 (3.6) |  | 2.14 (0.96–4.75) | 2 (1.6) | 57 (4.1) |  | 0.39 (0.09–1.64) | 3 (6.7) | 57 (3.6) |  | 0.97 (0.22–4.22) |
| Mental stress | |  |  |  |  |  |  |  |  |  |  |  |  |
| N (%) | Low | 83 (83.0) | 1,235 (86.4) |  | - | 96 (77.4) | 1,205 (85.9) |  | - | 36 (80.0) | 1,326 (84.8) |  | - |
|  | High | 17 (17.0) | 195 (13.6) |  | 1.52 (0.87–2.66) | 28 (22.6) | 198 (14.1) |  | 1.87 (1.17–2.97) * | 9 (20.0) | 237 (15.2) |  | 1.57 (0.70–3.54) |

*OR* odds ratio, *NRS* numerical rating scale, *95% CI* ninety-five percent confidence interval.

* Significant at the 0.05 level.

^a^ Crude estimate. ^b^ Adjusted for gender, continuous body mass index, continuous age, physical workload, daily smoking and mental stress. ^c^ *Low* indicates sedentary work, *medium* ambulatory work, and *high* activities such as heavy lifting and climbing.
